# High density lipoprotein (HDL) particles from end-stage renal disease patients are defective in promoting reverse cholesterol transport

**DOI:** 10.1038/srep41481

**Published:** 2017-02-02

**Authors:** Josephine L.C. Anderson, Thomas Gautier, Niels Nijstad, Markus Tölle, Mirjam Schuchardt, Markus van der Giet, Uwe J.F. Tietge

**Affiliations:** 1 Department of Pediatrics, University Medical Center Groningen, University of Groningen, Groningen, The Netherlands; 2 INSERM UMR866 Lipides, Nutrition, Cancer; Faculté de Médecine, Dijon, France; 3 Medizinische Klinik IV – Nephrology, Charite – Campus Benjamin Franklin, Berlin, Germany

## Abstract

Atherosclerotic cardiovascular disease (CVD) represents the largest cause of mortality in end-stage renal disease (ESRD). CVD in ESRD is not explained by classical CVD risk factors such as HDL cholesterol mass levels making functional alterations of lipoproteins conceivable. HDL functions in atheroprotection by promoting reverse cholesterol transport (RCT), comprising cholesterol efflux from macrophage foam cells, uptake into hepatocytes and final excretion into the feces. ESRD-HDL (n = 15) were compared to healthy control HDL (n = 15) for their capacity to promote *in vitro* (i) cholesterol efflux from THP-1 macrophage foam cells and (ii) SR-BI-mediated selective uptake into ldla[SR-BI] cells as well as (iii) *in vivo* RCT. Compared with HDL from controls, ESRD-HDL displayed a significant reduction in mediating cholesterol efflux (p < 0.001) and SR-BI-mediated selective uptake (p < 0.01), two key steps in RCT. Consistently, also the *in vivo* capacity of ESRD-HDL to promote RCT when infused into wild-type mice was significantly impaired (p < 0.01). *In vitro* oxidation of HDL from healthy controls with hypochloric acid was able to fully mimic the impaired biological activities of ESRD-HDL. In conclusion, we demonstrate that HDL from ESRD patients is dysfunctional in key steps as well as overall RCT, likely due to oxidative modification.

Plasma levels of high density lipoprotein (HDL) cholesterol are strongly inversely correlated with the risk of atherosclerotic cardiovascular disease (CVD) in populations with normal kidney function^1^,^2^. The beneficial effects of HDL are largely ascribed to the role of HDL in reverse cholesterol transport (RCT), i.e. the transport of excess cholesterol from the periphery back to the liver for excretion into bile^2^,^3^,^4^. For efficient RCT two steps are of critical importance, (i) cholesterol efflux from macrophage foam cells mainly mediated by ABCA1 and ABCG1^2^,^5^ and (ii) SR-BI-dependent cholesterol uptake into hepatocytes^2^,^6^.

CVD represents the single largest cause of morbidity and mortality in patients with reduced kidney function or uremia, reflected by a 30-fold increase in age-adjusted CVD mortality in end-stage renal disease (ESRD) patients^7^,^8^. Although a number of classical as well as non-classical risk factors have been reported to contribute to this excessive increase in CVD mortality, the underlying pathophysiological basis for these observations is still insufficiently understood^9^. Chronic kidney disease itself might not result in a substantial impairment of the cholesterol efflux function of HDL^10^, while HDL from patients on hemodialysis exhibits an apparent reduction in the capacity to accept cholesterol from macrophages^11^,^12^,^13^,^14^. However, the ability of ESRD-HDL to function in the whole RCT pathway has not been investigated thus far.

Therefore, the present study not only tested the functional properties of HDL from ESRD patients for the two major steps of RCT *in vitro*, namely cholesterol efflux from macrophages and SR-BI-mediated cholesterol delivery but also the ability of ESRD-HDL to promote RCT from ^3^H-cholesterol-loaded macrophages *in vivo* in mice. Our results indicate that ESRD-HDL is less efficient than control HDL in mediating RCT, conceivably due to oxidative modifications of HDL apolipoproteins.

## Results

### HDL from ESRD patients displays defective cholesterol uptake as well as cholesterol delivery properties *in vitro*

**Figure 1:**
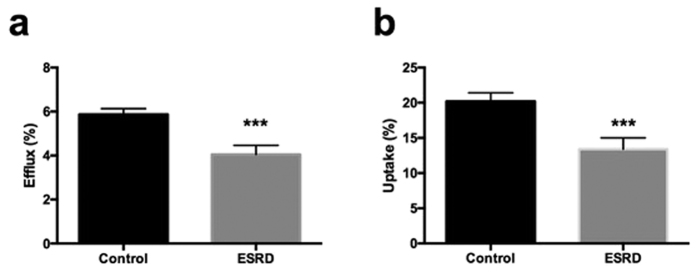
ESRD-HDL displays defective cholesterol uptake as well as cholesterol delivery properties. (**a**) Cholesterol efflux from primary mouse peritoneal macrophages towards HDL from ESRD-patients (n = 15) compared with healthy control subjects (n = 15). (**b**) Cellular SR-BI mediated selective cholesterol uptake from ESRD-HDL or control HDL (each n = 15) into ldla cells stably transfected with SR-BI. Data are presented as means ± SEM. ***p < 0.001.

Two important functional properties enable HDL to serve as an efficient mediator of RCT, namely (i) to elicit cholesterol efflux from macrophage foam cells and (ii) to deliver cholesterol to cells via the SR-BI-mediated selective uptake pathway. To test these properties for HDL from ESRD patients we first performed cholesterol efflux experiments. Compared with HDL from control subjects, ESRD-HDL displayed a significant reduction in mediating cholesterol efflux (5.87 ± 0.26 vs. 4.05 ± 0.41%, p < 0.001, Fig. 1a). Next, we tested the capacity of ESRD-HDL to deliver cholesterol into ldla cells stably transfected with SR-BI. Also cellular cholesterol uptake from ESRD-HDL via SR-BI was significantly impaired for HDL from ESRD patients compared with controls (20.2 ± 1.2 vs. 13.4 ± 1.6%, p < 0.001, Fig. 1b). These data demonstrate that ESRD-HDL is defective in both properties crucial for functional RCT.

### HDL from ESRD patients displays an altered lipid and protein composition

Compared with controls, HDL particles from ESRD patients were significantly enriched in triglycerides (Supplemental Table S2, p = 0.001). While the cholesteryl ester content of HDL was decreased in ESRD patients (p < 0.05), free cholesterol content was increased (p < 0.05). Phospholipid and protein contents did not differ between the two experimental groups. Regarding HDL proteins associated with impaired functionality of the particle we found ESRD HDL significantly enriched in both serum amyloid A (SAA, 27.4 ± 5.5 vs. 3.1 ± 0.5 μg/dl, p < 0.001) and apoC-III (10.3 ± 0.7 vs. 8.1 ± 0.8 mg/dl, p < 0.05).

In ESRD patients, HDL cholesterol was positively associated with both the efflux (r = 0.58, p < 0.05) and selective uptake function (r = 0.53, p < 0.05), while no correlations were detected with the HDL triglyceride content. In addition, SAA within HDL correlated inversely to efflux (r = −0.71, p < 0.01) but not significantly to selective uptake (r = −0.43, p = 0.11). No correlations were found with the apoC-III content of HDL and either efflux (r = −0.26, n.s.) or selective uptake (r = −0.12, n.s.).

### HDL from ESRD patients is defective in mediating RCT *in vivo*

Since HDL from ESRD patients showed impaired cholesterol uptake and delivery properties *in vitro*, we next tested the functional behaviour of ESRD-HDL in an integrated *in vivo* physiological setting of RCT. Mice that had received macrophage foam cells loaded with radioactively labeled cholesterol were infused with either PBS, control HDL or ESRD-HDL, and appearance of the tracer in different compartments was followed over time.

**Figure 2:**
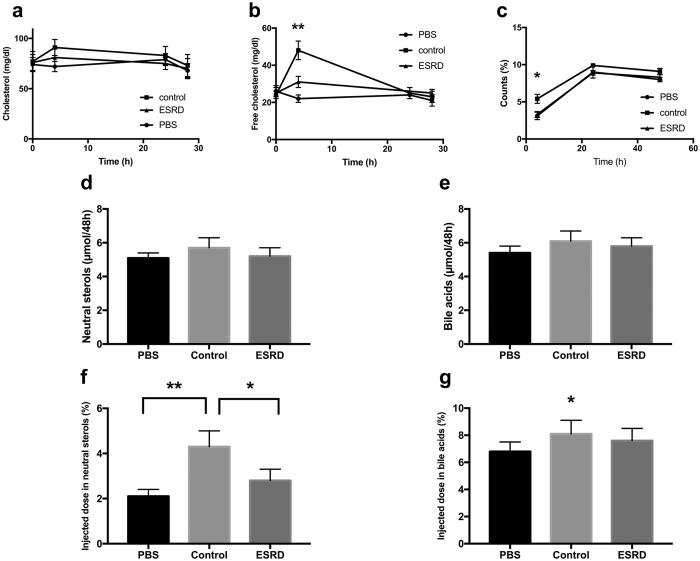
HDL from ESRD patients are defective in medicating RCT *in vivo*. Mice injected with macrophage foam cells loaded with radioactively labelled ^3^H-cholesterol were infused with either PBS, control HDL or ESRD-HDL as detailed in methods. (**a**) Mass changes in plasma total cholesterol, (**b**) mass changes in plasma free cholesterol, (**c**) appearance of the ^3^H-cholesterol tracer in plasma, (**d**) mass fecal neutral sterol excretion over 48 h, (**e**) mass fecal bile acid excretion over 48 h, (**f**) fecal ^3^H-cholesterol tracer excretion within neutral sterols, (**g**) fecal ^3^H-cholesterol tracer excretion within bile acids. Data are presented as means ± SEM, n = 6–8 mice/group. *p < 0.05, **p < 0.01.

First, we assessed mass changes in cholesterol in the plasma compartment in response to the different treatments. As shown in Fig. 2a, only the group receiving control HDL exhibited an increase in plasma total cholesterol at the early 4 h time point, essentially due to significantly higher plasma free cholesterol levels (p < 0.01, Fig. 2b). The group receiving the ESRD-HDL was not different in these parameters from PBS controls. In agreement with the mass data also appearance of macrophage-derived ^3^H-cholesterol in plasma was only higher at the 4 h time point in the control-HDL group (p < 0.05, Fig. 2c). These data are consistent with ESRD-HDL having a reduced capacity to elicit cholesterol efflux *in vivo* analogous to our *in vitro* findings.

At the 48 h time point livers from PBS injected mice contained significantly less macrophage-derived ^3^H-cholesterol (2.97 ± 0.10%) than livers from mice infused with either control HDL (4.10 ± 0.23%, p < 0.05) or ESRD-HDL (3.78 ± 0.14%, p < 0.05), while there was no difference between control and ESRD-HDL receiving groups.

Cholesterol can either be excreted from the body within the fecal neutral sterol fraction or after metabolic conversion to bile acids. The mass fecal excretion of neutral sterols (Fig. 2d) and bile acids (Fig. 2e) did not change upon the different treatments. On the other hand, control HDL resulted in a significant increase in tracer excretion within neutral sterols (Fig. 2f) as well as within bile acids (p < 0.05 for ESRD, p < 0.01 for PBS, Fig. 2g) causing an overall substantial increase in RCT. However, ESRD-HDL failed to have any significant effect on the fecal exretion of the macrophage-derived ^3^H-cholesterol, indicating that the *in vivo* capacity to mediate effective RCT is significantly impaired in these particles in comparison to HDL from healthy controls.

### Oxidation of control HDL *in vitro* results in impaired cholesterol uptake as well as delivery properties

**Figure 3:**
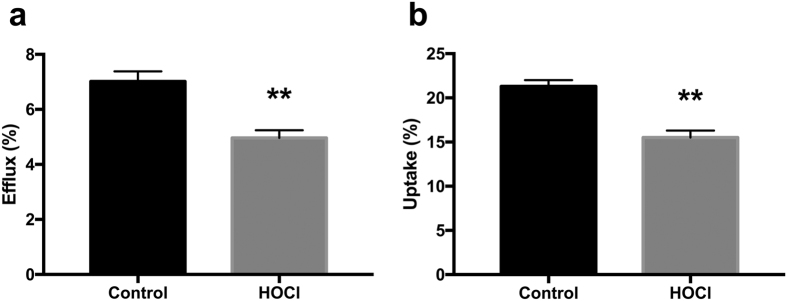
Oxidation of HDL *in vitro* results in impaired cholesterol uptake as well as delivery properties. (**a**) Cholesterol efflux from primary mouse peritoneal macrophages towards native, control HDL compared with control HDL following HOCl incubation (n = 10). (**b**) Cellular SR-BI mediated selective cholesterol uptake form control HDL or HOCl-modified HDL (n = 10) into ldla cells stably transfected with SR-BI. Data are presented as means ± SEM. **p < 0.01.

Since ESRD patients show a substantial increase in inflammatory load and oxidative stress^15^,^16^,^17^, we speculated that a possible mechanism underlying the decreased RCT functionality of ESRD-HDL might be oxidation of apolipoproteins contained within the HDL particle, which are of crucial importance to its function. Therefore, we next determined TBARS levels within HDL as a measure of oxidative modification. While in control HDL TBARS were detectable at a considerably low level, all ESRD-HDL tested contained substantial amounts of TBARS consistent with our hypothesis (0.7 ± 0.1 vs. 4.2 ± 0.6 nmol/mg, p < 0.001). In addition, TBARS content of HDL correlated negatively with the two functional parameters determined in our study, namely cholesterol efflux (r = −0.58, p = 0.02) and selective uptake (r = −0.52, p < 0.05). Further, we aimed to test the pathophysiological consequences of HDL oxidation on the two functional properties important for RCT, cholesterol uptake and delivery. HDL oxidatively modified by incubation with HOCl displayed a significantly reduced capacity to serve as acceptors for macrophage-mediated cholesterol efflux compared with control HDL (7.02 ± 0.36 vs. 4.96 ± 0.28%, p < 0.01, Fig. 3a). In addition, also the SR-BI-mediated uptake of cholesterol from oxidized HDL was significantly decreased (21.3 ± 0.7 vs. 15.5 ± 0.8%, p < 0.01, Fig. 3b). These data show that *in vitro* oxidatively modified HDL are defective in both properties, mediating cholesterol efflux and delivering cholesterol to cells via SR-BI, comparable with the functional deficits observed for ESRD-HDL.

### HDL oxidized *in vitro* is defective in mediating *in vivo* RCT

**Figure 4:**
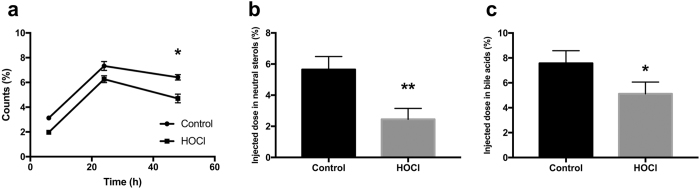
HDL oxidized *in vitro* is defective in mediating *in vivo* RCT. Mice injected with macrophage foam cells loaded with ^3^H-cholesterol were infused with either control HDL or HOCl-modified HDL as detailed in methods. (**a**) HOCl-modified HDL had a significantly decreased capacity to mobilize macrophage-derived ^3^H-cholesterol to the plasma compartment compared with native HDL at the 4 h time point (p < 0.05). (**b**) Fecal ^3^H-cholesterol tracer recovery in the neutral sterol fraction, (**c**) Fecal ^3^H-cholesterol tracer recovery in the bile acid fraction. Data are presented as means ± SEM, n = 8 mice/group. *p < 0.05, **p < 0.01.

Next, we tested the *in vivo* functionality of oxidized HDL in RCT. HOCl-modified HDL had a significantly decreased capacity to mobilize macrophage-derived ^3^H-cholesterol to the plasma compartment compared with native HDL at the 48 h time point (p < 0.05, Fig. 4a). In addition, counts recovered in the fecal neutral sterol fraction (p < 0.01, Fig. 4b) as well as in the fecal bile acid fraction (p < 0.05, Fig. 4c) were significantly lower with HOCl-modified HDL, indicating that oxidative modification of healthy control HDL substantially decreases its *in vivo* capacity to function in RCT.

## Discussion

Combined, the results of this study demonstrate that HDL from patients with ESRD is dysfunctional in mediating RCT, a key atheroprotective property^2^,^3^, conceivably due to extensive oxidative modifications of HDL associated proteins. Reduced RCT is thus expected to contribute to the excessive increase in CVD risk observed in ESRD patients.

A progressive reduction in kidney function is known to associate with a significant increase in CVD risk^7^,^8^,^18^. This relationship culminates in an approximately 30-fold increase in age-adjusted CVD risk in ESRD patients^7^,^8^. Plasma HDL cholesterol levels in ESRD patients are decreased^19^,^20^, however, to our knowledge the ability of HDL particles from ESRD patients to function in overall RCT has not been investigated thus far. In the present study we used in addition to *in vitro* studies an *in vivo* approach to directly measure RCT from macrophages to feces^3^,^5^,^6^,^21^. Our data thereby add a reduction in the RCT functionality of HDL to pathophysiological concepts of increased CVD in patients with reduced kidney function and uremia.

An interesting question is the underlying pathophysiology and therefore the mechanistic basis for the oxidative modifications observed in this study within HDL apolipoproteins in ESRD. It is established that ESRD patients display a proinflammatory state and suffer from an increased oxidative stress burden^22^,^23^,^24^. Myeloperoxidase (MPO) is an enzyme expressed by macrophages and neutrophils that is released in response to proinflammatory stimuli^25^. Interestingly, plasma levels of MPO have been shown to be significantly increased in patients with ESRD and also to be a predictor of mortality in hemodialysis patients^26^,^27^. MPO has been previously demonstrated to cause oxidative modifications of HDL apolipoproteins that might impact their functionality^25^,^28^. Hypochloric acid (HOCl) can mimic MPO-induced oxidation *in vitro*. By modifiying HDL from healthy controls with HOCl we were able to replicate the functional deficits displayed by ESRD-HDL, namely a decreased efficacy in promoting cholesterol efflux as well as a reduced ability to deliver cholesterol to cells via SR-BI. These data provide an additional line of evidence that MPO might play a key role in causing the decreased functionality of HDL in ESRD and that MPO might therefore represent an interesting target for pharmacological inhibition in ESRD patients.

Testing HDL functionality in addition to measuring mass HDL cholesterol and apoA-I levels is an emerging concept in the field of HDL research^29^,^30^,^31^,^32^. However, although multiple potentially beneficial effects have been described for HDL^33^, there are limitations for the clinical setting, since reliable and reproducible assays to test these functions are lacking^30^. Thus far, several dysfunctionalities were ascribed to HDL from ESRD patients. It was reported that ESRD is less effective in protecting LDL against copper mediated oxidation *in vivo*^34^. These data could be interpreted as a further indication that HDL from ESRD patients is already oxidized to a substantial extent and therefore cannot properly fulfil anti-oxidative functions. Furthermore, a decreased functionality of HDL-mediated cholesterol efflux has been demonstrated in ESRD patients^11^,^35^. Regarding testing properties related to RCT *in vitro* our data suggest that also assays addressing selective uptake through SR-BI from given HDL preparations might be valuable, since this also represents a key step for effective RCT that might be differentially affected compared with cholesterol efflux.

For a balanced interpretation of our results the following points should be taken into account. (i) We demonstrate that ESRD-HDL is dysfunctional in mediating RCT in wild-type mice. RCT in mice largely depends on HDL and the HDL selective uptake receptor SR-BI. In contrast, in humans RCT is mainly based on the LDL receptor mediating hepatic cholesterol uptake following transfer of cholesteryl esters out of HDL into apoB-containing lipoproteins by the cholesteryl ester transfer protein (CETP), which is not expressed in rodents^36^. Although ESRD-HDL apparently has a decreased interaction with several relevant components of the HDL metabolism system, it remains to be formally established that also in humans ESRD-HDL is defective in mediating *in vivo* RCT. (ii) In addition, we focussed in our *in vitro* studies on the classical RCT pathway, investigating macrophage efflux and hepatic uptake. We did not assess a contribution of transintestinal cholesterol excretion (TICE) to RCT, which might also have relevance here^37^. In previous work, we estimated the contribution of TICE to RCT to be around 30% under baseline conditions in wild-type mice^4^, while others found higher values^38^. However, results from lipoprotein kinetic studies indicate that HDL does not represent the lipoprotein subclass that donates cholesterol to the TICE pathway^4^,^39^, at least not directly. In a system expressing CETP such as humans, this contribution might be higher, but this questions remains to be experimentally answered. (iii) Furthermore, the patient and the control group differed in several aspects other than the presence/absence of ESRD such as diabetes, smoking or use of lipid lowering medication and we cannot formally exclude that these differences impact the results. However, there were no differences within the ESRD group between either smokers/non-smokers, diabetic/non-diabetic patients and patients with or without lipid lowering medication (data not shown). Furthermore, also the results for cholesterol efflux as well as selective uptake were not different between ESRD patients displaying all additional risk factors combined (smoking, diabetes, lipid lowering medication) and those without, while each ESRD group differed significantly from controls (Supplemental Figure S1). These data indicate that the presence of ESRD might have a substantially stronger effect on the impairment of HDL function than any of these potential confounders. Also, none of the conclusions changed when the smokers were excluded from analysis (data not shown). (iv) The design of our study does not enable us to draw a firm conclusion, if the observed differences in HDL function are due to the hemodialysis treatment or the presence of ESRD. Future studies including patients with ESRD naïve to hemodialysis are warranted to address this issue.

In summary, our data demonstrate that HDL from ESRD patients is extensively oxidatively modified and displays reduced efficacy in key protective functions related to CVD, namely promoting (i) cellular cholesterol efflux, (ii) SR-BI-mediated cholesterol delivery and (iii) overall functional RCT *in vivo*. These results might have major implications to explain the excessive increase in CVD risk in uremic patients.

## Materials and Methods

### Patients and control subjects

EDTA plasma was collected under fasting conditions from patients with ESRD and age- and sex-matched controls (n = 15 each, see Supplemental Table S1 for clinical and biochemical characteristics). Blood samples were placed on ice immediately after collections and stored at −80 °C until analysis. Patients and controls were in a stable clinical condition and free from infectious complications for at least 3 months. None of the ERSD patients had residual renal function. Blood samples of the ESRD group were taken before regular hemodialysis sessions. Informed consent was obtained from all subjects. Blood collection was approved by the responsible medical ethics committee of the Charité Berlin and methods were carried out in accordance with the approved guidelines.

### Cholesterol efflux experiments

Thioglycollate-elicited mouse peritoneal macrophages^40^ were loaded with ^3^H-cholesterol (1 μCi/ml, NEN Life Sciences Products), and 50 μg acetylated LDL for 22 h as described^14^ followed by equilibration in RPMI with 0.2% BSA for 18 h. Following another wash with PBS, acceptors were added (individual HDL samples isolated by ultracentrifugation as detailed below, 50 μg of protein, experiments were performed in triplicates). After 5 h the supernatant was taken off and radioactivity within the medium was determined by liquid scintillation counting (Beckman LS6500, Beckman Instruments, Palo Alto, CA). Next, 0.1 M NaOH was added to cells, plates were incubated for 30 min at room temperature and the radioactivity remaining within the cells was then also assessed by liquid scintillation counting. Efflux is given as the percentage of counts recovered from the medium in relation to the total counts present on the plate (sum of medium and cells). Values for unspecific efflux determined as release of ^3^H-cholesterol from macrophages in the absence of HDL were subtracted from the individual values.

### HDL uptake experiments

LdlA cells lacking LDL receptor expression as well as ldlA cells stably transfected with a murine SR-BI cDNA (ldlA[mSR-BI]) were kindly provided by Dr. Monty Krieger (MIT, Boston, USA) and cultured as described^41^. For HDL cholesterol uptake experiments, 5% lipoprotein-depleted serum was used. HDL was isolated from individual plasma samples as described below and labeled with cholesteryl hexadecyl ether (cholesteryl-1,2-^3^H, NEN Life Sciences Products), a non-hydrolyzable analogue of cholesteryl ester with identical selective uptake properties as ^3^H-cholesteryl ester, essentially as described^42^,^43^. Ten μg/ml of ^3^H-CE HDL was added to the cells, experiments were performed in duplicates. After a 5-h incubation, the cells were washed three times with PBS (pH 7.4) and lysed with 0.5 ml of 0.1 M NaOH. Tracer uptake was calculated as counts recovered from the cells as percentage of the total dose (counts from cells added to the counts from media).

### HDL composition analysis

For the analysis of HDL composition, HDL was isolated from 200 μl of plasma by tabletop sequential ultracentrifugation (1.063 < *d* < 1.21) as described^44^, and total and free cholesterol, phospholipids and triglycerides were determined enzymatically using commercially available reagents (Wako Pure Chemical Industries, Neuss, Germany). Protein concentrations were measured with the BCA assay (Pierce, Rockford, IL, USA). Commercially available ELISA kits were used according to the manufacturer’s instructions to determine human SAA (Biosupply, Bradford, UK) and apoC-III (Abcam, Cambridge, UK) in HDL.

### *In vivo* RCT

C57BL/6 J mice, 8 weeks of age, were purchased from Charles River (Sulzfeld, Germany). The animals were kept in animal rooms with alternating 12-hour periods of light (from 7:00 a.m. to 7:00 p.m.) and dark (from 7:00 p.m. to 7:00 a.m.), with ad libitum access to water and mouse chow diet (Arie Blok, Woerden, The Netherlands). Animal experiments were performed in accordance with the national laws. All protocols were approved by the responsible ethics committees of the Landesamt für Gesundheit, Ernährung und technische Sicherheit Berlin (LAGETSI) and the University of Groningen. Thioglycollate-elicited macrophages were isolated, cultured and loaded with AcLDL and ^3^H-cholesterol exactly as detailed above. At the end of the equilibration, macrophages were carefully harvested from the plate and injected intraperitoneally into mice (2 × 10E6 cells/mouse). Directly following the i.p. injection of the macrophages, individually housed mice were administered i.v. either PBS (200 μl) or pooled HDL (200 μl) either from the ESRD patients group or from the group of control subjects at a dose of 2 mg HDL cholesterol/mouse. The choice to base injections on HDL cholesterol was made to be able to detect potential functional differences of HDL independent of the established clinical biomarker HDL cholesterol, whose validity has recently been called into question^36^. Injections were repeated at 24 h. Blood samples were drawn at time points 0 h, 4 h, 24 h and 48 h and tracer within plasma was determined by liquid scintillation counting. After 48 h, mice were sacrificed, the liver was harvested and total feces produced over the 48 h period of the experiment were collected.

Radioactivity in plasma was counted directly, counts for ^3^H-cholesterol taken up by the liver were determined by incubating a piece of liver with Solvable (Packard, Meriden, CT, USA) according to the manufacturer’s instructions to dissolve the tissue as previously published^45^. Counts recovered from a respective piece of liver were backcalculated to total liver mass and expressed as percent of injected dose per whole organ. Feces were thoroughly dried, ground and aliquots were separated into the bile acid and the neutral sterol fractions^46^. Counts recovered from the respective aliquots were related to the total amount of feces produced within 24 h and expressed as percentage of the injected radiotracer dose.

### *In vitro* oxidative modification of HDL

Oxidized HDL was generated following a previously published procedure^47^. Briefly, 1 mg/ml of total HDL protein isolated from healthy controls (1.063 < *d* < 1.21) was incubated with NaOCl solution at a molar ratio of 200 for 60 min at 37 °C, adjusted to a final pH of 7.4. Preparations of modified HDL were dialyzed against PBS and used within 24 h. For uptake experiments, oxidized HDL were labeled with cholesteryl hexadecyl ether as described above.

### Statistical analysis

Statistical analyses were performed using the statistical package for social sciences (SPSS; SPSS Inc., Chicago, IL). Data are presented as means ± SEM. Statistical differences between two groups were assessed using the Mann-Whitney U-test. To compare more than two groups ANOVA followed by a Bonferroni post-hoc test was used. Statistical significance for all comparisons was assigned at P < 0.05.

## Additional Information

**How to cite this article**: Anderson, J. L.C. *et al*. High density lipoprotein (HDL) particles from end-stage renal disease patients are defective in promoting reverse cholesterol transport. *Sci. Rep.* **7**, 41481; doi: 10.1038/srep41481 (2017).

**Publisher's note:** Springer Nature remains neutral with regard to jurisdictional claims in published maps and institutional affiliations.

## Supplementary Material

Supplementary Information
