# Supplementary material for: High density lipoprotein (HDL) particles from end-stage renal disease patients are defective in promoting reverse cholesterol transport
Source: Sci Rep. 2017 Feb 2;7:41481. doi: 10.1038/srep41481 (PMC5288657; doi:10.1038/srep41481)
Supplement: Supplementary Information [file srep41481-s1.pdf]

Supplementary Information:

High density lipoprotein (HDL) particles from end-stage renal disease patients are defective in promoting reverse cholesterol transport

Josephine L.C. Anderson[1], Thomas Gautier[2], Niels Nijstad[1], Markus Toelle[3], Mirjam Schuchardt[3], Markus van der Giet[3], and Uwe J.F. Tietge[1]

[1] Department of Pediatrics, University Medical Center Groningen, University of Groningen, Groningen, The Netherlands

[2] INSERM UMR866 Lipides, Nutrition, Cancer; Faculté de Médecine, Dijon, France

[3] Medizinische Klinik IV – Nephrology, Charite – Campus Benjamin Franklin, Berlin, Germany

Supplemental Table S1: Clinical and biochemical characteristics of the end-stage renal disease (ESRD) patients and controls investigated

	ESRD (n=15)	Controls (n=15)	*P* value
Age (years)	48 (31-56)	46 (26-64)	n.s.
Sex (male/female)	10/5	9/6	n.s.
Body weight (kg)	74 (56-83)	69 (59-90)	n.s.
Duration of dialysis (months)	38 (24-78)	0	
Serum creatinine (mg/dl)	7.1 (5.4-9.4)	1.0 (0.7-1.2)	<0.001
Blood urea nitrogen (mg/dl)	26 (22-38)	14 (10-16)	<0.001
Total protein (g/l)	64 (50-81)	69 (67-73)	n.s.
Cholesterol (mg/dl)	251 (184-283)	180 (166-214)	<0.05
CRP (mg/l)	5.8 (2.0-8.5)	1.1 (0.7-1.5)	<0.001
Smoking	3 (20)	0 (0)	<0.01
Diabetes	6 (40)	0 (0)	<0.001
Coronary heart disease	6 (40)	0 (0)	<0.001
Lipid lowering drugs	9 (60)	0 (0)	<0.001

Data are presented as median (range) or total numbers (%).

Supplemental Table S2: Mass composition of HDL isolated from ESRD patients and controls

	controls (n=15)	ESRD (n=13)
Cholesterol ester (%)	14.1 ± 0.3	11.3 ± 0.9§
Free cholesterol (%)	4.5 ± 0.1	4.8 ± 0.1§
Phospholipids (%)	14.5 ± 0.6	13.7 ± 0.7
Triglycerides (%)	2.8 ± 0.1	5.2 ± 0.5§
Protein (%)	64.2 ± 0.7	65.0 ± 1.1

Values are means ± SEM.

Significantly different from controls: § at least $p<0.05$.

Supplemental figure S1

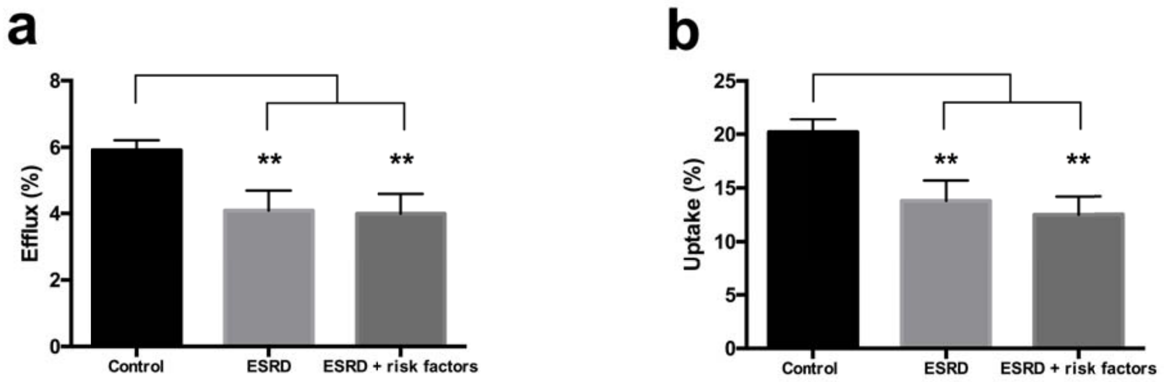

Supplemental figure S1: ESRD-HDL displays defective cholesterol uptake as well as cholesterol delivery properties. (a) Cholesterol efflux from primary mouse peritoneal macrophages towards HDL and (b) Cellular SR-BI mediated selective cholesterol uptake form HDL. In these experioments HDL was used isolated either from healthy controls subjects (control, n=15), ESRD-patients without additional risk factors (ESRD, n=5) and ESRD patients with at least one of the additional risk factors smoking, diabetes and lipid lowering medication (ESRD + risk factors, n=10). Data are presented as means ± SEM. *$p<0.05$, ***$p<0.001$
